# Numerical and Experimental Study into Paper Compression Test

**DOI:** 10.3390/ma16247513

**Published:** 2023-12-05

**Authors:** Leszek Czechowski, Paweł Pełczyński, Maria Bieńkowska, Włodzimierz Szewczyk

**Affiliations:** 1 Department of Strength of Materials, Lodz University of Technology, Stefanowskiego Street 1/15, 90-537 Lodz, Poland; 2 Centre of Papermaking and Printing, Lodz University of Technology, Wolczanska Street 221, 93-005 Lodz, Poland; pawel.pelczynski@p.lodz.pl (P.P.); maria.bienkowska@p.lodz.pl (M.B.); wlodzimierz.szewczyk@p.lodz.pl (W.S.)

**Keywords:** paper strength, digital image analysis, finite element method, Hill’s anisotropy potential theory

## Abstract

The study aims to present the results of paper compression under an axial load. Different heights of samples subjected to compression were taken into account. The main goal of the analysis was to determine experimentally the maximum compression load. In addition, numerical models based on the finite element method (FEM) were validated to refer to empirical results. The performed numerical simulations were founded on Green–Lagrangian nonlinear equations for large displacements and strains. The progressive failure of the compressed orthotropic material after exceeding maximum stresses was based on Hill’s anisotropy theory. Nonlinear calculations were conducted by using a typical Newton–Raphson algorithm for achieving a sequence convergence. The accuracy of the developed model was confirmed experimentally in compression tests. The technique of analysing the shape of the compressed paper sample on the basis of images recorded during the measurement was used. The obtained test results are directly applicable in practice, especially in the calculation of the mechanical properties of corrugated cardboard and in determining the load capacity of cardboard packaging. Knowing the maximum compressive stress that packaging paper can withstand allows packaging to be properly designed and its strength assessed in the context of the transport and storage of goods.

## 1. Introduction

Paper is an orthotropic material widely used for the production of corrugated and cellular cardboard, from which a variety of packaging is created [1]. The strength properties of these packages depend to a large extent on the properties of the paper. Much effort is devoted to researching these properties of paper. Authors of [2] explore the material’s response to compressive forces. They enhance our understanding of its structural integrity under pressure. Reference [3] sheds light on the complex phenomenon of creep in corrugated board, which is a key aspect when packaging fresh produce. This research provides valuable information on the challenges of maintaining product integrity during transportation and storage. The research presented in [4] integrates experimental and numerical approaches to comprehensively study paperboard tube failure. The synergy of methods enhances the robustness of findings, contributing to a deeper understanding of lateral stress impacts on paper structures. The work [5] introduces a novel method for assessing the radial crush strength of paper cores; this source marks a notable advancement in evaluating paper strength properties. The innovative approach presented holds promise for redefining industry standards in the assessment of paper core integrity. Study [6] presents a comprehensive approach, examining the crushing dynamics of double-wall corrugated cardboard and its impact on the load-bearing capacity of boxes. This allows us to look at packaging materials from an energy perspective. In [7], the response of a paper honeycomb to changing relative humidity was investigated. This helps us to understand how environmental conditions affect the stresses of these structures. Works [8,9,10] acknowledge the significance of environmental conditions, specifically humidity, in influencing the properties of honeycomb structures. However, paper [11] introduces a new method for measuring the edgewise compression properties of paper. It laid the groundwork for understanding the fundamental compression characteristics of paper, serving as a historical reference in the evolution of testing methodologies. On the other hand, work [12] presents a modelling framework for understanding both global and local buckling phenomena in corrugated board panels subjected to edge-to-edge compression. The research sheds light on the structural intricacies of corrugated materials under compression, contributing to the broader understanding of their mechanical behaviour. There is a need to minimize the weight of the packaging while maintaining the required strength. To achieve this, it is increasingly customary to predict the strength properties of packaging by calculating the properties of the papers used in its production [13,14,15,16,17,18]. One of the most important properties is the resistance to edge crushing, which is measured in a short-plug compression test known as an SCT [19,20]. However, the SCT test does not take into account the phenomenon of buckling of the sample, which occurs at longer fastening lengths and is commonly observed under the operating conditions of corrugated packaging. The phenomenon of sample buckling causes a significant reduction in its load capacity and it is necessary to take it into account in the prediction of this quantity [21,22,23,24,25,26,27]. Thanks to modern methods of processing and analysis of digital images [28,29,30,31], it is possible to observe changes in the shape of paper samples during the test of resistance to crushing by compressive forces [32,33]. This allows the change in the shape of the sample to be linked to the force acting on it during the test. To achieve this, images of the tested sample are recorded. As a result, it is possible to observe and evaluate the buckling parameters of the sample, in particular its shape and the size of the deflection arrow. The results of the image analysis of the paper sample compression process provide valuable data for numerical prediction of their load capacity. This allows for a more effective search for a numerical model describing the process of paper destruction in a unidirectional compression test.

The research presented in this article focused on the development and validation of a numerical model describing the process of crushing paper with compressive forces acting in its plane on the basis of experimental data from a testing machine and resulting from the analysis of a sequence of sample images recorded during the measurement [34]. Thanks to this approach, it was possible to verify the prediction of the shape of the paper sample subjected to the test, which is a strong confirmation of the validity of the developed model. Moreover, taking into account Green–Lagrangian nonlinear equations for large displacements and strains, progressive failure based on Hill’s anisotropy potential theory was assumed to validate the numerical model and to achieve numerically maximum loads.

## 2. Materials and Methods

### 2.1. Measuring Stand

**Figure 1:**
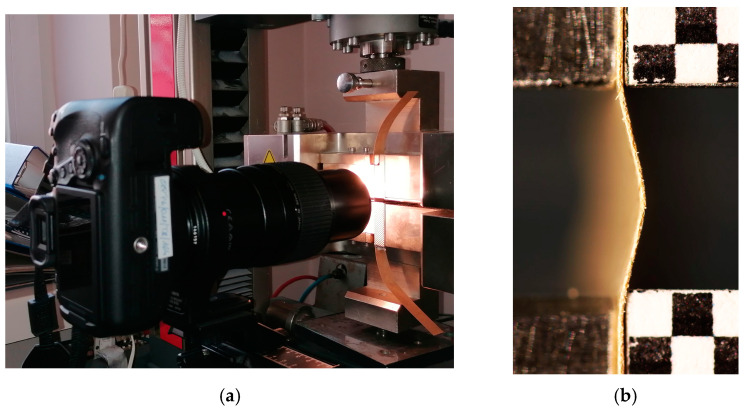
The measurement setup composed of UTM and DSLR camera (**a**) and UTM handles with chessboard patterns glued to them and a paper sample under test (**b**).

The SCT test of paper samples is usually made using a universal testing machine (UTM). This allows us to record the shortening of the sample and the accompanying force. To enable the assessment of the shape of the sample during the measurement, the sample was recorded using a vision system based on a single camera. In order to synchronize the shape registration of the paper sample subjected to the test and the measurement of the displacement of the movable UTM handle, a measuring station as shown in Figure 1a was developed. The stand is a modification of a measurement setup developed for registering the local deformation of paper in a one-directional tensile test [35]. It consists of a Zwick Roell Z 010 testing machine (89079 Ulm, Germany) with SCT test holders and a camera. The design of the holders allows for much longer connection lengths than in a standard SCT. Thanks to this, it was possible to perform the presented experiments and observe the phenomenon of buckling of paper samples. The camera used is a Canon EOS 6D Mark II digital SLR camera (Hong Kong, China) with a Canon MP-E 65 mm f/2.8 1-5x Macro lens. The camera is placed on a tripod that provides stable conditions for photo recording. The sample is illuminated by a white light source ensuring constant illumination. The camera is attached to the tripod via a setting shoe with the possibility of adjusting the position of the parallel and perpendicular axis to the optical axis of the camera. Chessboard patterns of a single square length of 1 mm (Figure 1b) are glued onto the handles of the testing machine. This allows us to track the movement of the handle and determine the spatial resolution of the recorded images. The measurement begins by placing the test paper sample in the holders of the testing machine and positioning the camera ensuring the visibility of the sample and fragments of chessboards in its field of view. The recording of the photo sequence is then triggered and, after recording several images, the measurement is initiated in the machine. Reaching the desired displacement of the movable handle completes the measurement. The number of recorded images depends on the duration of the compression test. The photo capture rate is 4 fps, but is not constant. In some cases, it dropped after the camera cache was full. Nevertheless, thanks to reading information from photo EXIF about the time of taking the photo with an accuracy of 1/100 of a second, it was possible to precisely synchronize the photos with the UTM measurement results. The measuring station and the measurement procedure are described in more detail in [34].

### 2.2. Image Processing and Analysis Technique

**Figure 2:**
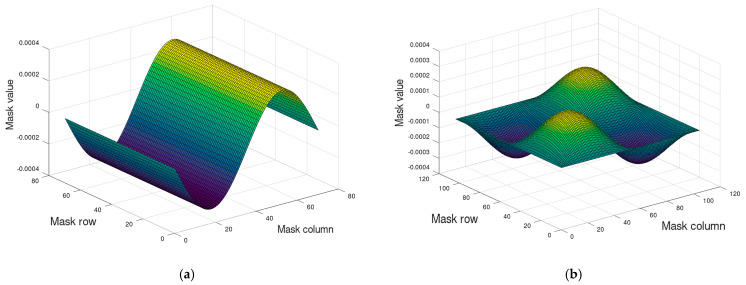
The shape (**a**) of the filter mask to emphasize the edges in the image; (**b**) the shape of the filter mask to emphasize the corners of the chessboard.

The recorded sequence of images was subjected to preprocessing and analysis. Pre-processing consisted of converting images to grayscale, extracting images of the examined paper sample, a chessboard on a movable handle and a chessboard on a fixed handle, linear filtering of the sample image using a filter highlighting vertical edges in the image with the mask shown in Figure 2a and linear filtering of chessboard images using a filter highlighting the corners of the chessboard corners in the image with the mask shown in Figure 2b.

**Figure 3:**
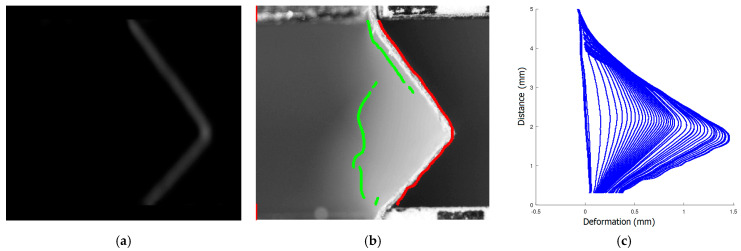
The result of the image filtering of the paper sample (**a**); the image of the sample with the edges detected (**b**); and the shape of the right edge of the tested sample obtained in subsequent images of the analysed sequence (**c**).

The image resulting from the filtering of the paper sample image is shown in Figure 3a. In Figure 3b, the original image of the sample with the left (green) and right (red), detected edges, is presented. The left edge is detected with low efficiency due to low contrast in the image. For further analysis, the right edge was selected.

Sets of coordinates of points of the detected edges and corners were input information to the algorithm for image data analysis. Image coordinates were converted to metric coordinates (mm) based on information about the spatial resolution of the images and the arbitrary place of origin of the coordinate system (related to the place where the sample is attached to the stationary holder). The shape of the edges of the tested sample in subsequent images of the recorded sequence is shown in Figure 3c. Next, the average values of the coordinates of the corners of the moving and stationary chessboard were calculated. They were used to determine the movement of the handles as a function of the time of recording individual photos. On the basis of this information, the time of image recording as a function of the measurement data acquisition time in the testing machine was estimated. The result was the synchronization of data recording times from two different sources.

### 2.3. Hill’s Anisotropic Potential Theory

Material properties of orthotropic paper were assumed: *E_x_* = 5600 MPa, *E_y_* = *E_z_* = 2450 MPa, *G_xy_* = *G_xz_* = *G_yz_* = 2120 MPa, Poisson’s ratio 0.3. Index *x* denotes the direction of compression. This means that moduli *E_x_* and *E_y_* represent the properties in longitudinal and perpendicular directions with regard to the compression direction, respectively. In order to model the plasticity of the analysed paper, Hill’s anisotropic criterion was applied [36,37,38] to describe the anisotropy material behaviour after exceeding the yield stress of paper. If this criterion was considered for isotropic hardening, the yield function could be determined by [36,37,38]:

(1) $$f(\sigma )=\sqrt{{\left\{\sigma \right\}}^{T}[M]\{\sigma \}}-\sigma_{0}({\bar{\varepsilon }}^{p})$$

where $\sigma_{0}$ and ${\bar{\varepsilon }}^{p}$ represent a reference yield stress and equivalent plastic strain, respectively. For the material with the three orthogonal planes of symmetry (sym.), the plastic compliance matrix [*M*] can be given as:

(2) $$[M]=\left[\begin{array}{cccccc} G+M & -H & -G & 0 & 0 & 0\\ & F+H & -F & 0 & 0 & 0\\ & & F+G & 0 & 0 & 0\\ & & & 2N & 0 & 0\\ & & & & 2L & 0\\ & & & & & 2M \end{array}\right]$$

The coefficients mentioned above, *F*, *G*, *H*, *L*, *M* and *N*, are the material constants and should be defined experimentally. These magnitudes are shown in the following form:

(3) $$F=\frac{1}{2}\left(-\frac{1}{R_{xx}^{2}}+\frac{1}{R_{yy}^{2}}+\frac{1}{R_{zz}^{2}}\right),\;G=\frac{1}{2}\left(\frac{1}{R_{xx}^{2}}-\frac{1}{R_{yy}^{2}}+\frac{1}{R_{zz}^{2}}\right),\;H=\frac{1}{2}\left(\frac{1}{R_{xx}^{2}}+\frac{1}{R_{yy}^{2}}-\frac{1}{R_{zz}^{2}}\right)\;L=\frac{3}{2}\left(\frac{1}{R_{yz}^{2}}\right),\;M=\frac{3}{2}\left(\frac{1}{R_{xz}^{2}}\right),\;N=\frac{3}{2}\left(\frac{1}{R_{xy}^{2}}\right)$$

The yield stress ratios can be determined with the use of the following expressions as below:

(4) $$R_{xx}=\frac{\sigma_{xx}^{y}}{\sigma_{0}},\;R_{yy}=\frac{\sigma_{yy}^{y}}{\sigma_{0}},\;R_{zz}=\frac{\sigma_{zz}^{y}}{\sigma_{0}},\;R_{xy}=\sqrt{3}\frac{\sigma_{xy}^{y}}{\sigma_{0}},\;R_{yz}=\sqrt{3}\frac{\sigma_{yz}^{y}}{\sigma_{0}},\;R_{xz}=\sqrt{3}\frac{\sigma_{xz}^{y}}{\sigma_{0}}$$

where ${\bar{\sigma }}_{ij}^{y}$ represents the yield stress for appropriate directions. In the case of the application of Hill’s criterion, it was assumed that the tangential modulus *E^t^* after coming to the limit strength was 56 MPa and subsequently stress ratios *R_ij_* amounted to: *R_xx_* = 1, *R_yy_* = *R_zz_* = 0.44 and *R_xy_* = *R_xz_* = *R_yz_* = 0.3. On the basis of one-directional tensile tests of the paper sample, the yield stress $\sigma_{0}$ was determined to be 36 MPa.

### 2.4. Finite Element Model

**Figure 4:**
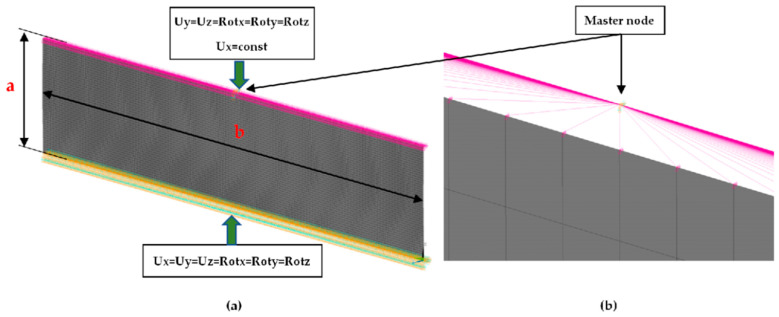
Model FE (**a**) and view of master node connected with outer nodes (**b**).

FE (finite element) calculations were carried out by using Ansys 18.2 software [38]. The dimensions *a* (0.7 mm, 1.3 mm, 2 mm, 2.5 mm, 3 mm, 3.5 mm, 4 mm, 4.5 mm, 5 mm) and *b* (15 mm) represent the height and the width of the sample, respectively (Figure 4). To elaborate adequate discrete models of the compressed samples, the 4-node 181-shell element was used. The size of finite element was assumed *a*/50. The buckling analysis (BA) was performed based on the linear block Lanczos algorithm to determine the critical loads. The nonlinear analysis was conducted for large strains and deflections on the basis of Green–Lagrangian equations. The number of substeps for the single calculation was assumed to be from 1000 up to 50,000. The maximum number of iterations for each substep was set up to 5000. Figure 4 shows the FE model and the boundary conditions. The compression load applied to the sample was realised by using a master node associated with slave nodes lying on the outer edges of the sample. The preliminary imperfection *w*_0_ of columns for the sake of considered model was assumed: 0.1 *t*, 0.5 *t*, 0.75 *t*, 1 *t*, 1.5 *t*, 2 *t*, 4 *t*, where t (0.14 mm) denotes the thickness of the sample. The initial deflection was related to the mode of the first buckling force of the analysed sample. Nonlinear estimations and convergence analysis were conducted by using the Newton–Raphson algorithm.

## 3. Results and Discussion

### 3.1. Shape of Paper Samples Subjected to Compressive Forces in Subsequent Measurement Phases

**Figure 5:**
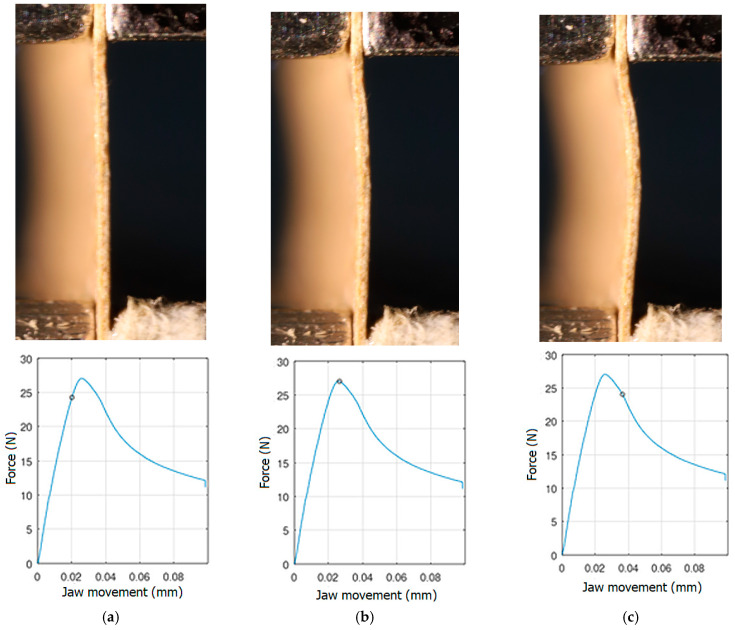
The shape of the paper sample and the corresponding point on the force-shortening curve for a clamping height of 3 mm at the beginning of buckling (**a**); at maximum force (**b**); at the end of the sine wave shape (**c**).

**Figure 6:**
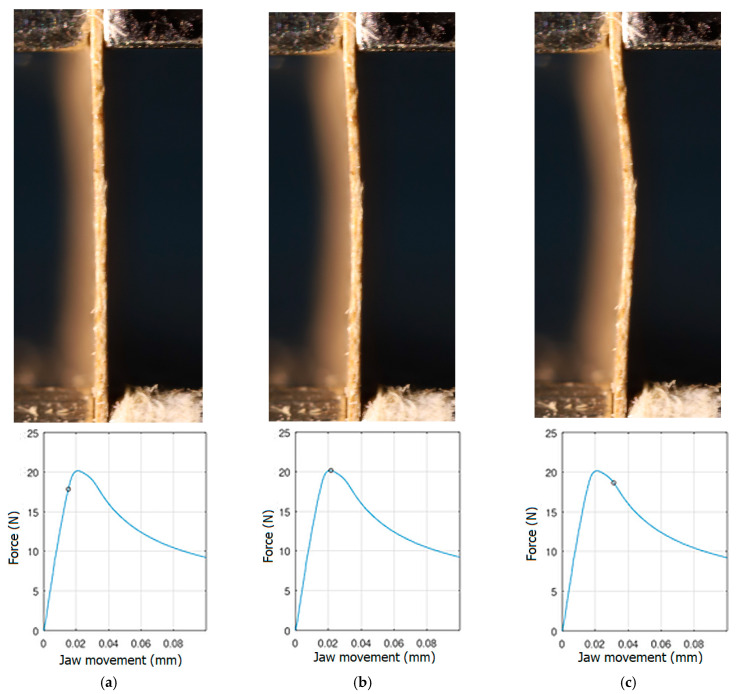
The shape of the paper sample and the corresponding point on the force-shortening curve for a clamping height of 4 mm at the beginning of buckling (**a**); at maximum force (**b**); at the end of the sine wave shape (**c**).

**Figure 7:**
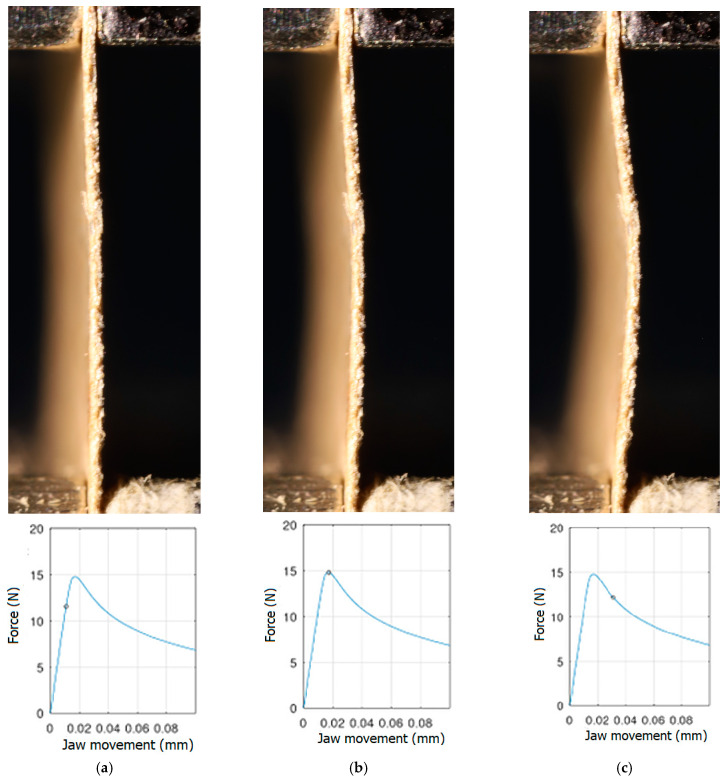
The shape of the paper sample and the corresponding point on the force-shortening curve for a clamping height of 5 mm at the beginning of buckling (**a**); at maximum force (**b**); at the end of the sine wave shape (**c**).

In order to be able to compare the results of paper modelling in compression tests with the results of real measurements in the UTM, the shapes of samples in measurement phases characterized by a change in sample behaviour were presented. The moment when the buckling started, the moment when the maximum compressive force was reached, and the moment when the sample broke and its shape of the buckled sample ceased to resemble a fragment of a sinusoid, were selected. Images of the samples are shown in Figure 5, Figure 6 and Figure 7.

The analysis of the recorded images reveals the occurrence of four phases of destruction of the samples, characterized by the following aspects:

1. compression without visible deflection—linear dependence of force on shortening;
2. significant deflection (possible appearance of buckling force) and consequent nonlinear increase in force with further shortening;
3. visible deformation of sample and nonlinear drop of force vs. shortening;
4. appearance of a joint in the middle of the sample or destruction in the area of the joints.

Figure 5, Figure 6 and Figure 7 show the shape of the samples and the corresponding points on the force- shortening curves at the boundaries between the mentioned destruction phases.

### 3.2. Critical Forces

This subsection shows the results of critical load calculations for a compressed paper sample with clamped supports (Figure 4). The estimations were performed for several heights a taking into account the first five buckling loads. Critical forces expressed in a Newton unit have been inserted in Table 1. Based on the results, it can be easily seen that for the shortest *a*, the critical forces are the greatest. It is interesting that the next critical loads are very close to each other. This means that the orthotropic material allows us to attain different modes with a small changing a force. In Table 2, total deformation maps are shown. Taking a look at the deformation modes, the number of half-waves for the appropriate buckling load are the same regardless of the considered height. This means that a small orthotropy (in the case of the analysed paper two to three times) does not matter significantly. Furthermore, these shown shapes at the application of the initial deflection were assumed.

### 3.3. Full Curves of Compression

**Figure 8:**
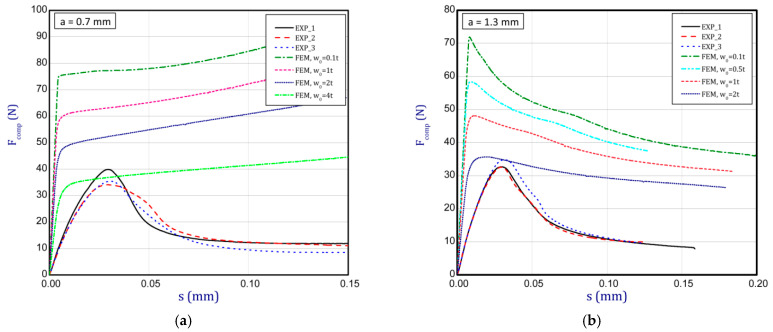
Shortening (*s*) vs. compression force (*F_comp_*) for height of 0.7 mm (**a**) and 1.3 mm (**b**).

**Figure 9:**
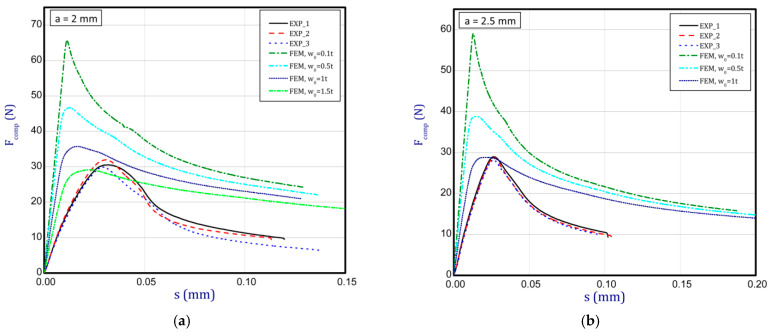
Shortening (*s*) vs. compression force (*F_comp_*) for height of 2 mm (**a**) and 2.5 mm (**b**).

**Figure 10:**
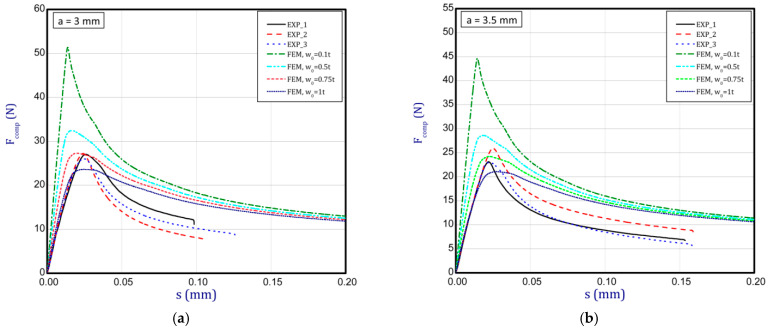
Shortening (*s*) vs. compression force (*F_comp_*) for height of 3 mm (**a**) and 3.5 mm (**b**).

**Figure 11:**
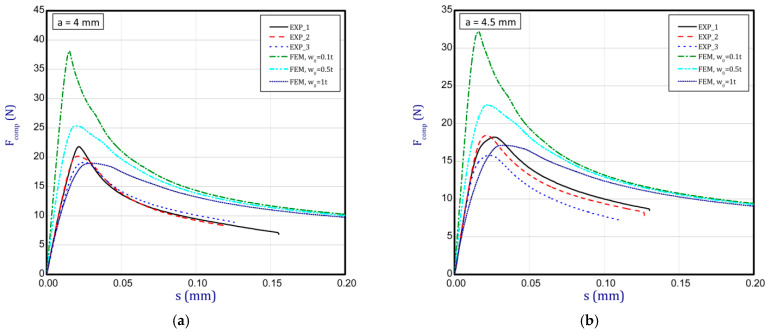
Shortening (*s*) vs. compression force (*F_comp_*) for height of 4 mm (**a**) and 4.5 mm (**b**).

**Figure 12:**
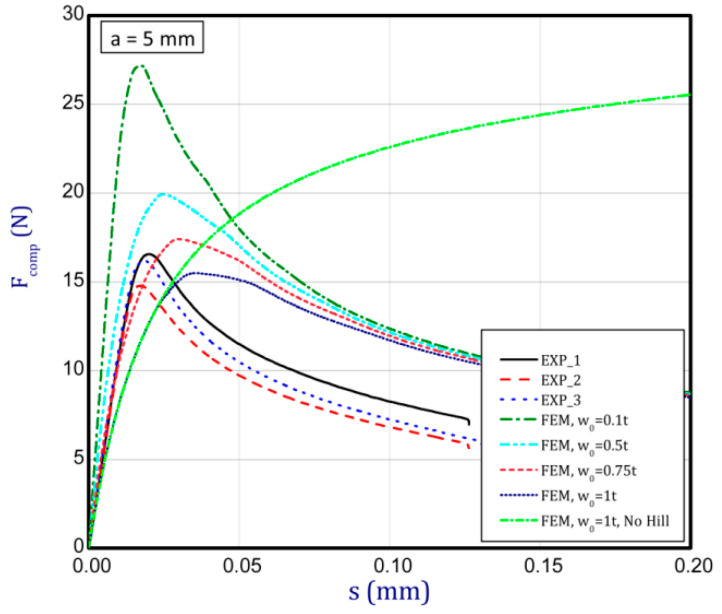
Shortening (*s*) vs. compression force (*F_comp_*) for height of 5 mm.

This subsection shows the diagrams of paper compression as the shortening s vs. the compression force *F_comp_* and gives maximum values of the forces. Based on the results, Figure 8a illustrates the curves of compression for the smallest considered height, i.e., *a* = 0.7 mm. The average peak of the force amounted to 38.2 N. It is 30 to 40 times smaller than the first critical force. If one can take into account the numerical curves, a significant divergence is seen in contrast with the experimental ones. Firstly, the stiffnesses (regardless of the magnitude of the initial imperfection) are meaningfully greater. Secondly, it was not possible to obtain maximum loads if even the initial deflection was equal to four thicknesses. However, it should be noted that at this variant the curves are the closest (between the experiment and the simulation). To justify this situation, one should note that the initial imperfection of the sample in the numerical model was based on modes achieved for linear buckling (See Table 2—for *a* = 0.7, first mode). Nevertheless, in the case of the experiment, the initial imperfection in this case could differ or be of another mode (through the whole width of the sample almost uniform curvature might have occurred, for example). This could mean that the resistance of paper under compression just for this variant caused a significant drop in stiffness and simultaneously a decrease in the maximum load that was observed in the experiment looking at registered curves. In the next graph (Figure 8b), if *a* = 1.3 mm was taken into consideration, in the numerical model some peaks were achieved. The mean maximum load in the experiment came to about 32 N. In the case of numerical estimations (for *w*_0_ = 2 *t*), the maximum load is 36 N. It can be seen that the curves go similarly, but in the case of the FE curve, the stiffness is still greater. By increasing the height *a*, the curves based on the FEA are getting closer to experimental curves. It is just observed on the next chart (Figure 9a) for *a* = 2 mm. Indeed, this effect is visible just for a greater preliminary deflection (between *w*_0_ = 1 *t* and *w*_0_ = 1.5 *t*). In the case of numerical curves, the maximum load is noticed at a smaller shortening (circa 0.01 mm, but for the experiment it is at 0.03 mm). Better consistency at the maximum load (experiment vs. simulation) is observed for height *a* = 2.5 mm (Figure 9b), because peaks amounted to 28 N for both cases (for *w*_0_ = 1 *t*). Looking through the next diagrams (Figure 10a,b, Figure 11a,b and Figure 12) where height a increases, it can be easily seen that there is better convergence between curves both in the maximum compression load and stiffness. Nevertheless, it should be admitted that initial imperfections used in numerical simulation are still great. In Figure 12, apart from the curves created based on Hill’s theory, for a comparison, the curve without application of this theory is shown. In this case, a different trend in the curve is seen after exceeding a limit stress (yielding of the paper might have been possible). Hence, this means that achieving a maximum load was not possible. The comparison of maximum loads obtained by both methods is set out in Table 3. Based on charts for greater *a*, it can be noted that within an increase in the initial deflection, both maximum loads and stiffnesses decrease; therefore, numerical curves begin to cover or be close to experimental ones.

### 3.4. Deformation Edge Points

**Figure 13:**
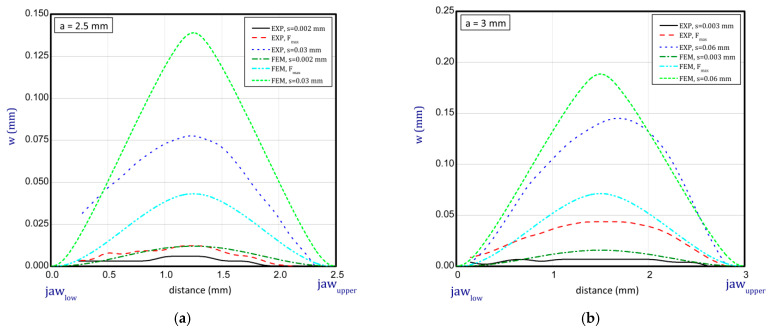
Deflection (*w*) vs. distance between jaws for height of 2.5 mm (**a**) and 3 mm (**b**).

**Figure 14:**
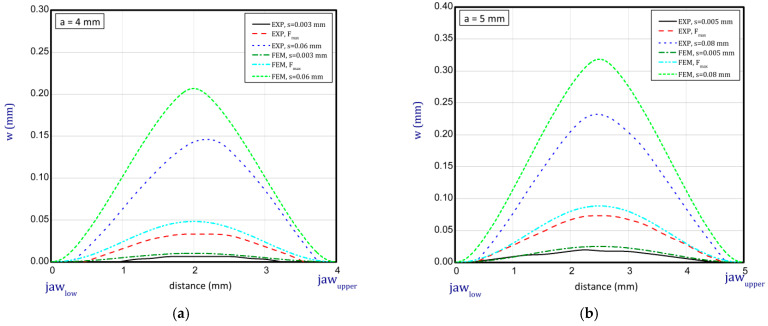
Deflection (*w*) vs. distance between jaws for height of 4 mm (**a**) and 5 mm (**b**).

This subsection shows some diagrams as a deflection *w* (growth of deflection) vs. the distance between the jaws for several stages (before buckling, at maximum load, at greater shortenings of sample) of compression for *a* = 2.5 mm, 3 mm, 4 mm and 5 mm (Figure 13 and Figure 14; for shorter samples, no buckling was observed). The deflections illustrated in the diagrams denote a displacement of the lateral edge of the sample. Based on these results, it can be seen that deflections obtained numerically are greater than in the experiment. For higher samples, these differences are not significant. Moreover, the modes in both methods seem to be similar. Of course, there are some discrepancies because peak points might have been lightly shifted with regard to the symmetry axis but in the experiment with regard to the imperfect structure such a behaviour of paper is possible and accessible. In the case of results for paper compression with *a* = 2.5 mm, the experimental displacements in sequential stages are not clearly visible as far as typical buckling modes are concerned. Therefore, the resistance of the paper is more noticeable because initial imperfections could be small and did not matter significantly. In general, the greater the lengths of samples that were considered, the better the convergences in deflections were achieved (e.g., for *a* = 4 mm or 5 mm).

## 4. Conclusions

The presented study was conducted to explore the compression behaviour of paper samples under an axial load, considering a few heights of samples. The SCT test assumed a pure compression without taking into account material buckling. However, this work involved the analyses both of buckling and its impact on the load-bearing capacity of paper. Through an empirical study, we determined the maximum compression loads and curves of compression whose results were the base of a validation of numerical models by using a finite element method, among others. The results of simulations, where numerical models were based on Green–Lagrangian nonlinear equations and Hill’s anisotropy theory, showed pretty good coincidence, especially for greater heights of compression; however, in the case of the smallest considered height, numerical determination of the maximum load was not possible even for a large initial imperfection. The reason this situation could result in an improper mode of initial deflection which finally might have been influenced by the entire stiffness. Nevertheless, the present study is the experimental confirmation of the developed model’s accuracy in compression tests, coupled with the innovative technique of shape analysis based on recorded images, and enhanced the practical applicability of the authors’ findings. These results directly benefit various industries, particularly in the evaluation of mechanical properties for corrugated cardboard and the determination of load capacity for cardboard packaging. Understanding the maximum compressive stress that packaging paper can withstand empowers the design of robust packaging solutions and aids in assessing their strength. Moreover, the conducted analysis of paper compression in the present paper allows us to explain and describe several phenomena occurring in paper goods. Firstly, one of the essential aspects can be obtaining different bending stiffnesses of corrugated cardboards for opposite signs of bending moments whose effect can be easily explained just by testing a deflection in the flat layer of cardboard taking into consideration the compression due to forces acting in their plane. Secondly, the performed tests of a paper compression determining the buckling effect and maximum loads may have defined the demanded lengths between the peaks in the wave (core) in multilayer corrugated cardboard.

## Figures and Tables

**Table 1:** The critical forces for different heights *a* between jaws.

	$F_{cr}$ (N)								
Mode	*a* = 0.7 mm	*a* = 1.3 mm	*a* = 2 mm	*a* = 2.5 mm	*a* = 3 mm	*a* = 3.5 mm	*a* = 4 mm	*a* = 4.5 mm	*a* = 5 mm
1	1124.3	415.3	187.5	122.2	85.7	63.4	48.7	38.6	31.3
2	1125.8	416.9	189.1	123.8	87.3	64.9	50.2	40.1	32.8
3	1128.4	420.2	192.6	127.4	90.9	68.6	54.0	43.9	36.7
4	1132.2	425.3	198.3	133.4	97.1	75.0	60.6	50.8	43.8
5	1137.4	432.5	206.5	142.1	106.3	84.7	70.8	61.4	54.9

**Table 2:** The buckling modes of compressed paper with several heights *a*.

a (mm)	Number of Mode				
	1	2	3	4	5
0.7	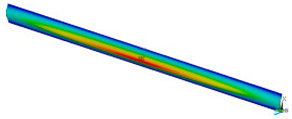	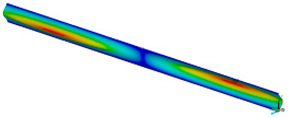	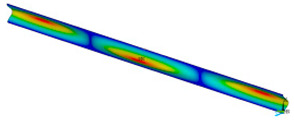	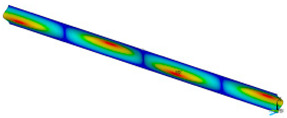	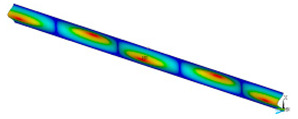
1.3	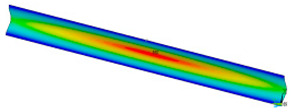	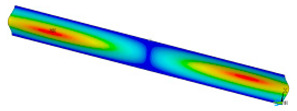	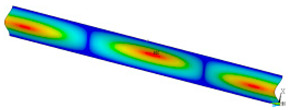	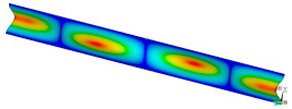	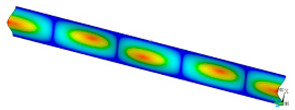
2	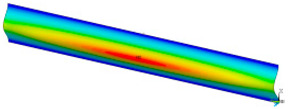	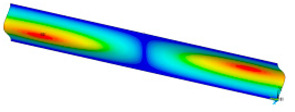	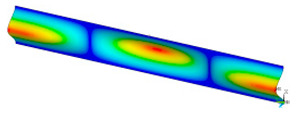	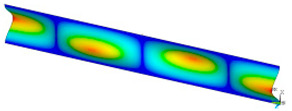	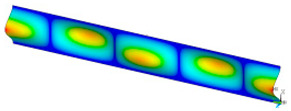
2.5	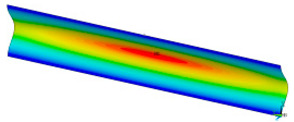	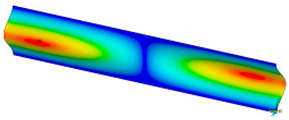	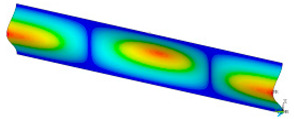	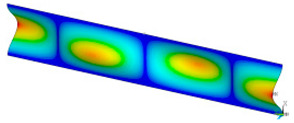	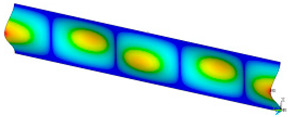
3	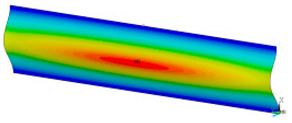	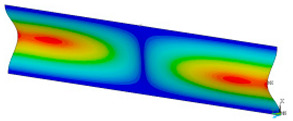	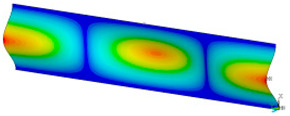	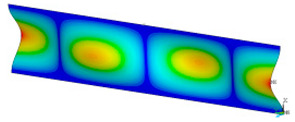	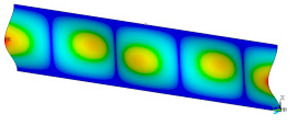
3.5	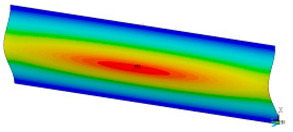	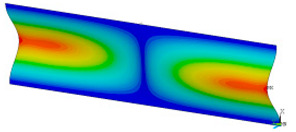	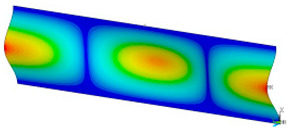	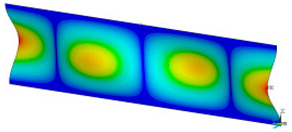	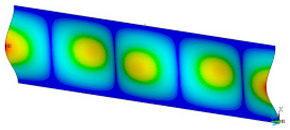
4	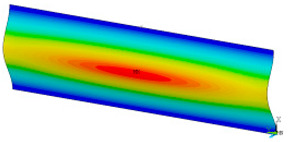	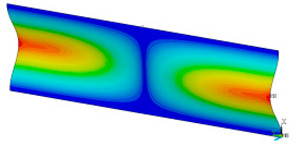	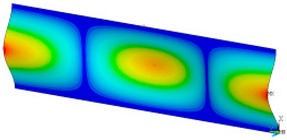	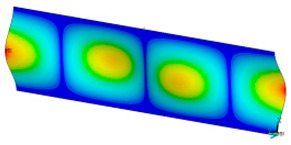	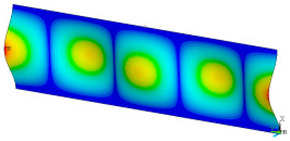
4.5	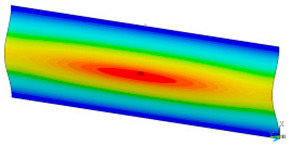	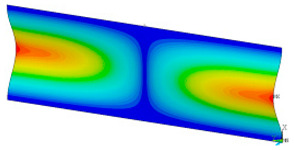	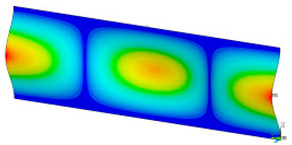	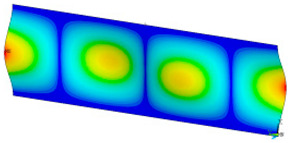	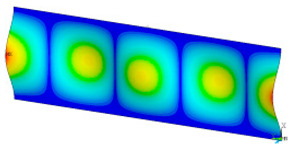
5	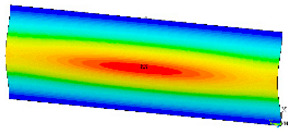	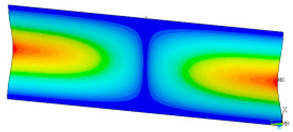	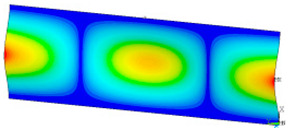	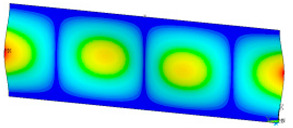	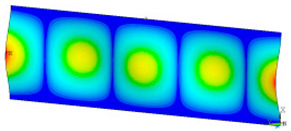

**Table 3:** The maximum compression forces for different length *a*.

	$F_{\max}$								
Variant	*a* = 0.7 mm	*a* = 1.3 mm	*a* = 2 mm	*a* = 2.5 mm	*a* = 3 mm	*a* = 3.5 mm	*a* = 4 mm	*a* = 4.5 mm	*a* = 5 mm
EXP_mean_	36.5 ± 3.1	33.4 ± 1.3	30.8 ± 1.0	28.7 ± 0.4	26.8 ± 0.7	24.0 ± 1.7	20.4 ± 1.4	17.5 ± 1.5	15.9 ± 0.9
FEM/ *w*_0_ = 0.1 *t*	-	71.9	65.7	59.3	51.6	44.8	38.2	32.2	27.2
FEM/ *w*_0_ = 0.5 *t*	-	58.3	46.7	38.8	32.4	28.6	25.3	22.5	19.9
FEM/ *w*_0_ = 0.75 *t*	-	-	-	-	27.3	24.2	-	-	17.4
FEM/ *w*_0_ = 1 *t*	-	48.1	35.7	28.8	23.6	21.1	19.0	17.1	15.5
FEM/ *w*_0_ = 1.5 *t*	-	-	29.1	-	-	-	-	-	-
FEM/ *w*_0_ = 2 *t*	-	35.6	-	-	-	-	-	-	-
FEM/ *w*_0_ = 4 *t*	-	-	-	-	-	-	-	-	-

## Data Availability

Data are contained within the article.
